# Incidence and spontaneous clearance of gonorrhea and chlamydia infections among men who have sex with men: a prospective cohort study in Zhuhai, China

**DOI:** 10.3389/fpubh.2024.1348686

**Published:** 2024-05-06

**Authors:** Hang Lyu, Haotong Tang, Yunlong Feng, Shuyan Hu, Yuyu Wang, Lanlan Zhou, Shanzi Huang, Jiarun Li, Huamei Zhu, Xi He, Weiming Tang, Yi Zhou, Lei Zhang

**Affiliations:** ^1^Zhuhai Center for Disease Control and Prevention, Zhuhai, China; ^2^Jieyang Center for Disease Control and Prevention, Jieyang, China; ^3^Longhua District Center for Disease Control and Prevention, Shenzhen, China; ^4^Fifth Affiliated Hospital of Zunyi Medical University, Zhuhai, China; ^5^Zhuhai Xutong Voluntary Services Center, Zhuhai, China; ^6^University of North Carolina Project-China, Guangzhou, China; ^7^Dermatology Hospital of Southern Medical University, Guangzhou, China; ^8^China-Australia Joint Research Center for Infectious Diseases, School of Public Health, Xi’an Jiaotong University Health Science Center, Xi’an, China; ^9^Artificial Intelligence and Modelling in Epidemiology Program, Melbourne Sexual Health Centre, Alfred Health, Melbourne, VIC, Australia; ^10^Central Clinical School, Faculty of Medicine, Nursing and Health Sciences, Monash University, Melbourne, VIC, Australia

**Keywords:** men who have sex with men, sexual behaviors, *Chlamydia trachomatis*, *Neisseria gonorrhoeae*, spontaneous clearance

## Abstract

**Background:**

Men who have sex with men (MSM) face significant risks of *Chlamydia trachomatis* (CT) and/or *Neisseria gonorrhoeae* (NG) infection. Nevertheless, only limited studies have looked into the site-specific infection and clearance of CT/NG. In order to prevent transmission, it is essential to understand the underlying factors that drive infection and spontaneous clearance.

**Methods:**

A 12-week cohort study examined the association between CT/NG infection, self-clearance, and sexual behaviors among MSM. The Willingness Service recruited participants who completed weekly questionnaires and provided urine, throat, and rectal swab samples.

**Results:**

The study involved 151 men, in which 51 (33.8%) were diagnosed with CT/NG infection during the study period. HIV (OR = 11.31), kissing (OR = 1.59), receptive oral sex (OR = 36.64), and insertive anal sex (OR = 19.73) constituted significant risk factors. 100% condom use (OR = 5.78) and antibiotic (OR = 7.53) were more likely to cause spontaneous clearance.

**Discussion:**

MSM may engage in riskier sexual behaviors due to insufficient knowledge and awareness of STI prevention, leading to increased susceptibility to NG/CT. It is crucial to concentrate on enhancing health education for MSM.

**Conclusion:**

This study found that the rectum was the most prevalent site of CT/NG and sexual behavior can influence the infection. Additionally, the appropriate use of antibiotics and consistent condom use may contribute to clear spontaneously.

## 1 Introduction

In recent decades, *Neisseria gonorrhoeae* (NG) and *Chlamydia trachomatis* (CT) have spread rapidly around the world, particularly among men who have sex with men (MSM) (1, 2). MSM has the characteristics of complex sexual partners and diverse high-risk behaviors. Anal or oral sex often leads to infection and transmission in the rectum and oropharynx. According to data from the general population, From 2009 to 2016, the estimated global prevalence of NG and CT in men was 0.7 and 2.7%, respectively, and in women, it was 0.9 and 3.8%, respectively, based on prevalence data from the general population (3). The prevalence of NG and CT in MSM was significantly higher than that in other populations. A study of MSM in the United States discovered that NG had a prevalence of 11.8%, whilst CT had a prevalence of 12.6% (4). In Kenya and Senegal, NG had a prevalence of 9.5 and 5.5%, respectively, among MSM and other gay men (5). The reported incidence of gonorrhea in China increased from 7.36/100000 in 2015 to 8.45/100000 in 2019 (6).

Epidemiological evidence suggests that both CT and NG can increase the sexual transmission of HIV infection (7). Studies have shown that the discharge of HIV in the secretions of people infected with HIV and CT is 3 times that of those not infected with HIV, which can increase the risk of HIV by 3 to 5 times (8). These sexually transmitted infections (STIs) have the potential to cause genital ulcers or mucosal inflammation, increasing the risk of transmission. Among MSM without HIV, both NG and CT infections are strong predictors of subsequent HIV infection. In 2011, the World Health Organization (WHO) recommended frequent and regular testing for asymptomatic urethral and rectal NG and CT infections for MSM using nucleic acid amplification techniques (9).

The elevated incidence of CT and NG among MSM can be ascribed to numerous factors. First, CT and NG are most commonly found among individuals aged between 20 and 39 years old, with those aged between 20 and 29 years old having the highest prevalence among MSM (9). Second, these infections are transmitted through sexual networks, where members have a history of multiple and concurrent sexual partnerships, which can amplify the spread of infections (2). Third, MSM are more likely to engage in high-risk sexual behaviors compared to men and women who have sex exclusively with women, such as having unprotected sex with multiple partners and engaging in other sexual practices like insertive anal sex and rimming. At the same time, there is a certain proportion of bisexual people in the MSM, which leads to the transmission of CT/NG infection not only in MSM, but also in the heterosexual population, resulting in adverse reproductive health outcomes for women (10, 11). Such sexual practices are significantly linked, and an epidemiological study that fails to acknowledge this correlation may be subject to confounding.

Chlamydia and gonorrhoea infections have been observed to resolve without antibiotic (1, 12). Several studies have found that spontaneous clearance of rectal-based CT infections can occur in women, MSM, and heterosexual men, ranging from 7 to 57%, while clearance of NG cases can occur in 20 to 33% of cases (12). Additionally, a retrospective study discovered that 11% of pharyngeal NG cases had cleared spontaneously. Factors such as bacterial load, age, and co-infection have been associated with spontaneous clearance of CT or NG (1, 13). Nevertheless, there is limited literature on the site-specific behavior and infection of MSM, and few studies have explored the site-specific infection and clearance of CT and NG or the connection between spontaneous clearance and previous infection, demographics, and sexual behaviors in the MSM population.

We conducted a prospective cohort study to identify different socio-demographic and sexual behavior factors that can affect the infection and clearance of CT/NG. We intensively collected data on sexual behavior and chlamydia and gonorrhoea infection status of 151 MSM over a 12-week follow-up period. The aim of this study is to analyze the factors associated with CT/NG infection and clearance, and to generate novel ideas for preventing STIs in MSM.

## 2 Methods

### 2.1 Study setting and participants

A prospective cohort study of MSM was conducted between November 29th, 2020, and October 25th, 2021, over a 12-week period for each participant. In total, we recruited 169 MSM at the Zhuhai Xutong Voluntary Services Centre (Zhuhai, China) and 151 individuals met the requirements of this study. The final visit of the last participant occurred on October 2nd, 2021. The inclusion criteria were (1) men who were 18 years or older, (2) born biologically male, (3) had sex with another man in the previous 3 months, (4) were willing to provide a contact number, and (5) agreed to participate in a follow-up survey in 12 weeks. Men who were unable to complete the 12-week follow-up were not eligible and 18 MSM were accordingly excluded from the study. MSM living with HIV were also eligible for the study.

### 2.2 Data collection

#### 2.2.1 Baseline data collection

At the baseline, the participants were requested to complete a baseline questionnaire, which included socio-demographic characteristics (including age, education level, nation, profession and residential locality), contact information, HIV and syphilis infection status (participants were tested for HIV and syphilis and informed of the results by our volunteers), the latest HIV detection time, sexual behavior information for the past 3 months (including the number of sex partners, the occurance and number of different sexual behaviors and the use of condoms), sexual behaviors sequence of the latest sexual activity, and the use of antibiotics for other purpose and mouthwash in the past 3 months. The sexual behaviors including kiss, receptive oral sex, insertive oral sex, receptive anal sex, insertive anal sex, receptive rimming, insertive rimming.

#### 2.2.2 Follow-up data collection

During follow-up, enrolled men were required to visit the hospital weekly for the duration of the study to be assisted by volunteers to collect swab samples, including urine sample, a pharyngeal swab and a rectal swab. The follow-up questionnaires were collected every week during the study period as well. The follow-up questionnaires included sexual behaviors information, sexual behavior sequence and the use of condom, antibiotics (for other purposes) and mouthwash in the past week.

#### 2.2.3 Sample collection

All baseline and follow-up questionnaires were collected via web-link on mobile devices in the presence of the study researcher. The collected biological samples that we collect were stored and tested in a single batch after 12 consecutive weeks of follow-up. Individuals who had positive diagnoses were subsequently informed and received further treatment. If any participants exhibit symptoms during follow-up, their biological sample will be tested with priority. If positive, the participants will be referred to further treatment and would not be allowed to return to the study until they are clear of the infection. All samples were kept at-80°C until the end of the study.

#### 2.2.4 Economic incentives

Throughout weeks 1–11 of the study’s follow-up period, participants were incentivized with a $1.50 subsidy for each sampling and completed questionnaire. When they completed the full 12-week study, participants were eligible to receive $250.00.

### 2.3 Laboratory testing

MSM participants provided oropharyngeal, urethral, and anal swabs over 12 weeks for *Chlamydia trachomatis* and Neisseria gonorrhoea molecular testing. The swabs underwent nucleic acid amplification testing (NAAT) using the Roche Amplicor kit (Roche Diagnostics, Branchburg, NJ, United States) to detect both pathogens. All samples were tested at the Fifth Affiliated Hospital of ZunYi Medical University in Zhuhai City, China.

### 2.4 Definitions of infection and spontaneous clearance

To ensure accuracy in our analysis, we defined chlamydia or gonorrhea infection as a participant having two consecutive weeks of a CT-or NG-positive specimen detected by NAAT in the same location (urethral, pharyngeal, or rectal) (14). Similarly, we defined chlamydia or gonorrhea spontaneous clearance as having two consecutive weeks of a CT-or NG-negative specimen after infection (1).

### 2.5 Statistical analysis

Statistical analysis was conducted using SPSS (Version 25, IBM, New York) and R Studio. Descriptive analyses were performed to evaluate participant outcomes. To compare socio-demographic characteristics, HIV and syphilis test results, and other behaviors of MSM between participants with chlamydia and/or gonorrhea infection and those without infection during the study, we utilized Chi-square test or Fisher’s Exact tests. For Cox analysis, we initially utilized univariate Cox proportional regression models to assess the relationships between risk factors and infection status. Afterwards, only the variables with a *p*-value less than 0.1 were incorporated into the multivariate Cox proportional regression models. The analysis factors include socio-demographic characteristics (age, educational level, nation, profession and place of residence), HIV and syphilis infection status, had ever had HIV testing, sexual behavior information (including the number of sex partners, the occurance and number of different sexual behaviors and the use of condoms), sexual behaviors sequence of the latest sexual activity (kiss, receptive oral sex, insertive oral sex, receptive anal sex, insertive anal sex, receptive rimming, insertive rimming), antibiotics and mouthwash use for other purpose within the past 3 months. *p* < 0.05 was considered statistically significant.

## 3 Results

### 3.1 Overall NG and CT infection

**Figure 1:**
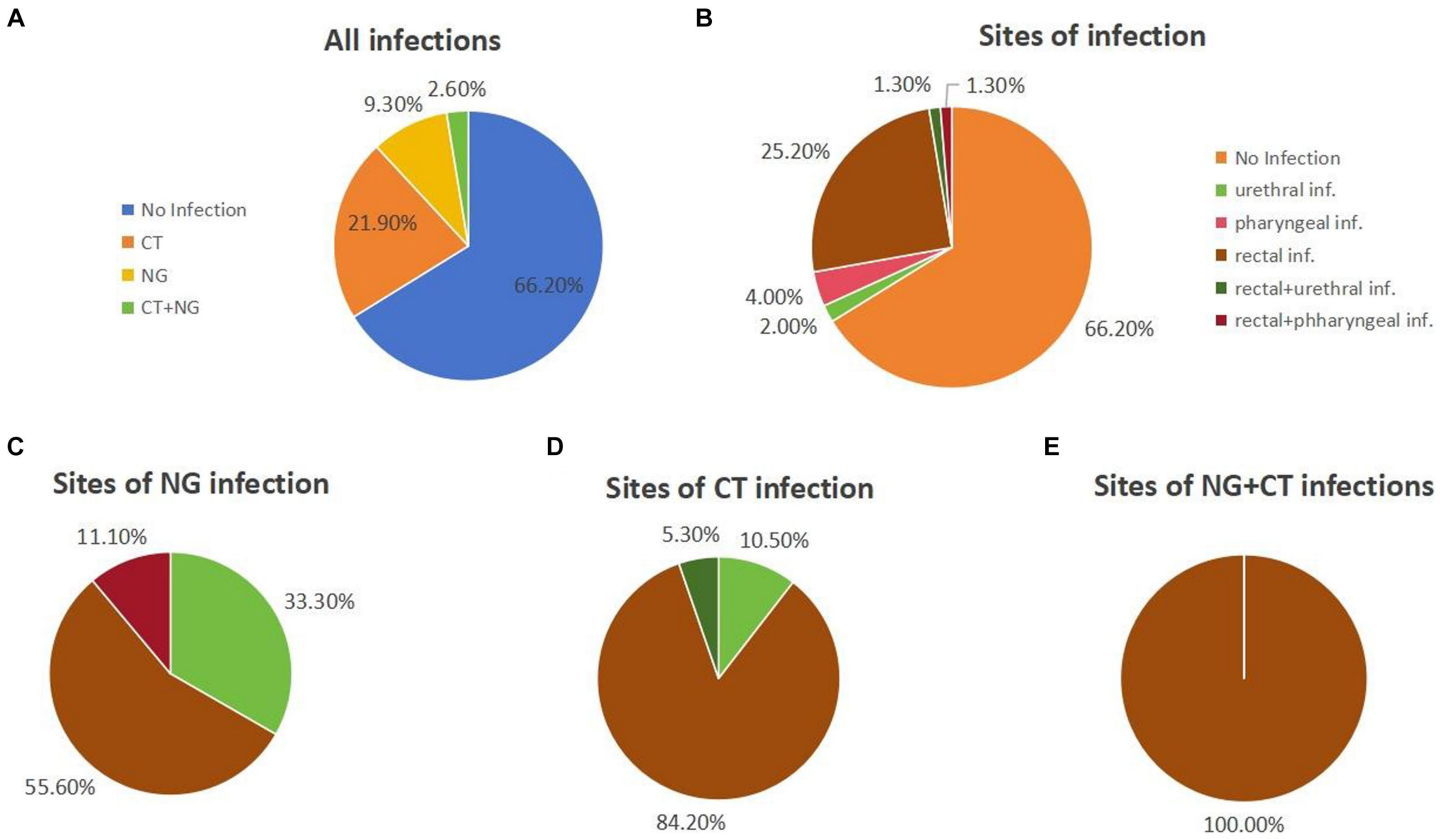
The statistics on chlamydia and/or gonorrhea infections during the 12-week follow-up. **(A)** Infections of chlamydia and/or gonorrhea in all individuals. **(B)** Sites of infection of all the individuals. **(C)** The sites of NG infection individuals. **(D)** The sites of CT infection individuals. **(E)** The sites of NG+CT infection individuals.

During the 12-week follow-up period, 51 out of 151 participants (33.8%) were found to have been infected with chlamydia and/or gonorrhea (Figure 1A). Of these, 21.9% (33/151) were infected with chlamydia only, 9.3% (14/151) were infected with gonorrhoea only. 4 participants (2.6%) tested positive for both chlamydia and gonorrhea (Figure 1A). Of all the participants, 25.2% (38/151) had a rectal infection, 4.0% (6/151) had a pharyngeal infection, 2.0% (3/151) had a urinary tract infection, and 3.0% (4/151) had two or more sites of infection (Figure 1B).

Among gonorrhoea-infected participants, 55.6% (10/18) had rectal infections, 33.3% (6/18) had urinary tract infections, and 11.1% (2/18) had both rectal and urinary tract infections (Figure 1C). As for the chlamydia-infected participants, 86.5% (32/37) had rectal infections, 8.1% (3/37) had pharyngeal infections, and 5.4% (2/37) had both rectal and pharyngeal infections (Figure 1D). All chlamydia and gonorrhoea co-infection occurred in the rectum (*n* = 4) (Figure 1E).

### 3.2 Characteristics of the participants

A total of 151 participants were recruited for the study. The baseline characteristics of the participants by population group and risk behaviors were summarized in Table 1. The median age of MSM was 29 years (IQR 25–33). The majority of the participants had a bachelor’s degree (89/151, 58.94%) and were office workers (70/151, 46.36%). In this study, most MSM was Han ethnicity (143/151, 94.70%) and resided in an urban city (125/151, 82.78%). Additionally, with regards to HIV testing, 96.03% (145/151) had HIV tests before. Among the participants, 2 out of 151 individuals (1.32%) tested positive for HIV. For syphilis status, 20.53% (31/151) got syphilis infection. Only 3.97% (6/151) had sex with a female before. In the past 3 months, 9.93% (15/151) MSM had used antibiotics for other purpose and most of the participants (83/151, 54.97%) never use mouthwash.

**Table 1:** MSM baseline demographic characteristics of 151 study participants.

		Total	CT/NG infection	No infection	*p* value
Variables		*N* = 151	*n* = 51	*n* = 100	
Age	18–24	35 (23.18)	18 (35.29)	17 (17.00)	0.06
	25–29	47 (31.13)	11 (21.57)	36 (36.00)	
	30–34	39 (25.83)	12 (23.53)	27 (27.00)	
	35 and older	30 (19.87)	10 (19.61)	20 (20.00)	
Education	Junior high and below	23 (15.23)	8 (15.69)	15 (15.00)	0.65
	High school	33 (21.85)	13 (25.49)	20 (20.00)	
	Bachelor’s degree	89 (58.94)	27 (52.94)	62 (62.00)	
	PhD or above	6(3.97)	3 (5.88)	3 (3.00)	
Nation	Han	143 (94.70)	46 (90.20)	97 (97.00)	0.08
	Other	8 (5.30)	5 (9.80)	3 (3.00)	
Residential location	City	125 (82.78)	41 (80.39)	84 (84.00)	0.77
	Town	17 (11.26)	6 (11.76)	11 (11.00)	
	Countryside	9 (5.96)	4 (7.84)	5 (5.00)	
Employment	Service	38 (25.17)	15 (29.41)	23 (23.00)	0.08
	Office worker	70 (46.36)	19 (37.25)	51 (51.00)	
	Individual entrepreneurship	14 (9.27)	6 (11.76)	8 (8.00)	
	Laborer	13 (8.61)	3 (5.88)	10 (10.00)	
	Student	10 (6.62)	7 (13.73)	3 (3.00)	
	Unemployed	6 (3.97)	1 (1.96)	5 (5.00)	
Had HIV test before	Yes	145 (96.03)	49 (96.08)	96 (96.00)	0.98
	No	6 (3.97)	2 (3.92)	4 (4.00)	
HIV status	Positive	2 (1.32)	2 (3.92)	0 (0)	**0.04**
	Negative	149 (98.68)	49 (96.08)	100 (100)	
Syphilis status	Positive	31 (20.53)	13 (25.49)	18 (18.00)	0.28
	Negative	120 (79.47)	38 (74.51)	82 (82.00)	
Had sex with a female*	Yes	6 (3.97)	2 (3.92)	4 (4.00)	0.98
	No	145 (96.03)	49 (96.08)	96 (96.00)	
Antibiotic use	Yes	15 (9.93)	6 (11.76)	9 (9.00)	0.59
	No	136 (90.07)	45 (88.24)	91 (91.00)	
Mouthwash use	At least twice a day	4 (2.65)	1 (1.96)	3 (3.00)	0.88
	Once a day	7 (4.64)	2 (3.92)	5 (5.00)	
	Multiple times a week	24 (15.89)	6 (11.77)	18 (18.00)	
	Once a week	2 (1.32)	1 (1.96)	1 (1.00)	
	Multiple times in the last 3 months	31 (20.53)	10 (19.61)	21 (21.00)	
	None in the last 3 months	83 (54.97)	31 (60.78)	52 (52.00)	

*For the past month. The significant of bold values *p* < 0.05.

Compared with no infection MSM, chlamydia and/or gonorrhoea infection participants were more likely to be HIV positive (*p* < 0.05) and young (*p* = 0.063).

### 3.3 Associations of factors with chlamydia and/or gonorrhoea infection

Table 2 shows the independent associations of socio-demographic characteristics with chlamydia and/or gonorrhoea infection. Relative to those nation Han, the multivariable-adjusted HRs (95% CIs) of chlamydia infection was 4.04 (1.56–10.43) for those nation other than Han. Relative to those HIV negative, the multivariable-adjusted HRs (95% CIs) of chlamydia infection was 8.38 (1.97–35.63) for those HIV positive. Age was found to be a protective factor against gonorrhoea infection in the multivariable-adjusted model (HRs: 0.89, 95% CI: 0.82–0.98). Additionally, compared to individuals who tested negative for HIV, those who tested positive exhibited a much higher risk of chlamydia and gonorrhoea infection (multivariable-adjusted HRs: 36.0, 95% CIs: 1.86–697.78).

**Table 2:** Hazard ratios and 95% confidence intervals for the associations between risk factors and infection status.

	CT infection				NG infection				CT/NG infection			
Variables	Univariate		Multivariate		Univariate		Multivariate		Univariate		Multivariate	
Nation	**3.8 (1.48–9.77)**	**0.01**	**4.04 (1.56–10.43)**	**0.00**	-	1.00						
Age	1 (0.95–1.05)	0.94			**0.9 (0.81–0.98)**	**0.02**	**0.89 (0.82–0.98)**	**0.02**	0.64 (0.39–1.07)	0.09	**0.71 (0.40–1.23)**	0.22
Education	0.6 (0.31–1.14)	0.12			1.06 (0.4–2.82)	0.910			-			
Residence location	1.38 (0.63–3.02)	0.42			1.36 (0.45–4.14)	0.585			-			
Have been infected with STI in the past 12 months	0.9 (0.4–2.05)	0.80			0.47 (0.11–2.04)	0.31			3.75 (0.23–59.92)	0.35		
HIV infection	**7.41 (1.76–31.26)**	**0.01**	**8.38 (1.97–35.63)**	**0.00**	5.72 (0.76–43.06)	0.09	6.36 (0.83–48.69)	0.08	**77.8 (4.83–1252.52)**	**0.00**	**36.0 (1.86–697.78)**	**0.02**
Syphilis infection	1.28 (0.6–2.71)	0.52			1.52 (0.54–4.27)	0.43			3.75 (0.23–59.92)	0.35		

The significant of bold values *p* < 0.05.

Table 3 presents the independent associations between sexual behavior factors and chlamydia or gonorrhoea infection. The number of recipient anal sex partners is significantly associated with chlamydia or gonorrhoea infection. Compared to those without a sex partner, individuals with one, two, three, and four or more sex partners had multivariable-adjusted HRs (95% CIs) of chlamydia infection of 1.94 (0.67–5.60), 2.45 (0.92–6.54), 5.20 (1.80–15.01), and 3.90 (1.50–10.12), respectively. Compared to those with no partners, the respective multivariable-adjusted HRs (95% CIs) for gonorrhea infection were 2.47 (0.50–12.23), 0.75 (0.08–7.25), 13.70 (3.26–57.55), and 6.47 (1.62–25.88) for individuals who engaged in anal sex as recipients with 1, 2, 3, and ≥ 4 partners.

**Table 3:** Multivariate cox regression for the associations between behavior and CT/NG infection.

	CT infection				NG infection			
Variables	Univariate		Multivariate		Univariate		Multivariate	
	OR (95% CI)	*p*	OR (95% CI)	*p*	OR (95% CI)	*p*	OR (95% CI)	*p*
**Frequency of sexual behavior**	1.05 (0.96–1.15)	0.28			1.02 (0.87–1.19)	0.82		
**Steps of sexual behavior**	0.85 (0.65–1.10)	0.21			0.93 (0.66–1.32)	0.70		
Kissed with male partner*	0.36 (0.05–2.65)	0.32			**-**	1.00		
Did you suck your male partner’s genitals*	1.38 (0.54–3.54)	0.50			1.04 (0.3–3.58)	0.96		
Did your male partner sucked your genitals*	0.78 (0.28–2.21)	0.64			0.52 (0.15–1.81)	0.307		
Did you lick your male partner’s anus*	1.25 (0.63–2.48)	0.53			1.27 (0.48–3.39)	0.63		
Did your male partner lick your anus*	0.87 (0.46–1.67)	0.68			0.74 (0.29–1.87)	0.53		
Had an anal sex with a male partner* (insertive)	0.77 (0.35–1.69)	0.52			1.04 (0.3–3.6)	0.95		
Had an anal sex with how many male partner* (recipient)		**0.01**		**0.02**		**0.00**		**0.00**
**0**	1.00		1.00		1.00		1.00	
**1**	1.94 (0.67–5.60)	0.22	1.87 (0.65–5.39)	0.25	2.47 (0.50–12.23)	0.27	2.47 (0.50–12.23)	0.27
**2**	2.45 (0.92–6.54)	0.07	2.27 (0.85–6.06)	0.10	0.75 (0.08–7.25)	0.81	0.75 (0.08–7.25)	0.81
**3**	**5.20 (1.80–15.01)**	**0.00**	**5.40 (1.86–15.71)**	**0.00**	**13.70 (3.26–57.55)**	**0.00**	**13.70 (3.26–57.55)**	**0.00**
**≥4**	**3.90 (1.50–10.12)**	**0.01**	**3.30 (1.27–8.61)**	**0.02**	**6.47 (1.62–25.88)**	**0.01**	**6.47 (1.62–25.88)**	**0.01**
Use condoms when sex*		0.06		0.06		0.40		
**Not 100% condoms**	1.00		1.00		1.00			
**Not had sex**	–	0.99	–	0.99	–	0.99		
**100% condoms**	0.55 (0.28–1.07)	0.08	0.50 (0.27–1.07)	0.08	1.48 (0.58–3.82)	0.42		

*In past 7 days. The significant of bold values *p* < 0.05.

### 3.4 Contributing factors to spontaneous clearance

*Chlamydia trachomatis* and *Neisseria gonorrhoeae* infections were found to have the capability of spontaneous clearance based on this cohort study. We defined spontaneous clearance as having negative test results for two consecutive weeks after a confirmed infection lasting for two consecutive weeks. Of the 52 diagnosed patients with chlamydia and/or gonorrhoeae infection, self-clearance occurred in 33 participants (63.5%). During the follow-up period, a total of 35 instances of spontaneous clearance were observed, of which 20 were chlamydia infection and 15 were gonorrhoea infection. Using condoms 100% of the time as insertive (OR: 5.78, 95% CI: 1.32–25.26) and having used antibiotics for other purposes (OR: 7.53, 95% CI: 2.39–23.64) in the past 7 days during follow-up were found to be associated with a higher likelihood of spontaneous clearance, as shown in Table 4. Furthermore, age seems to be a protective factor in regard to spontaneous clearance (OR:0.91, 95%CI: 0.82–1.00, *p* = 0.06).

**Table 4:** Factors associated with self-clearing between infected and self-clearing.

Variables	B	OR	OR (95% Cl)	*p-*value
**Age**	−0.10	0.91	**(0.82,1.00)**	**0.06**
**Education**				0.41
Lower education		1.00		
Medium education	1.14	3.13	(0.39,24.92)	0.28
Higher education	1.20	3.32	(0.55,19.91)	0.19
**Residence location**	−0.89	0.41	(0.03,5.08)	0.49
**Have you had two or more different sexual partners in the past month**	0.73	2.07	(0.32,13.29)	0.44
**Syphilis Infection**	−0.57	0.57	(0.12,2.61)	0.47
Have you ever kissed with your male partner*	−0.43	0.65	(0.24,1.76)	0.40
Have you sucked your male partner’s genitals*	−0.25	0.78	(0.27,2.19)	0.64
Had your male partner sucked your genitals*	0.22	1.25	(0.38,4.01)	0.71
Did you lick your male partner’s anus*	−0.47	0.62	(0.12,3.23)	0.58
Did your male partner lick your anus*	0.15	1.16	(0.29,4.59)	0.83
Have you ever had an anal sex with a male partner* (insertive)	−0.58	0.56	(0.12,2.45)	0.44
Have you ever had an anal sex with a male partner* (recipient)	−0.95	0.39	(0.09,1.63)	0.20
**Did you use condoms (insertive)**				**0.03**
Not 100% condoms		1.00		
100% condoms	1.76	5.78	**(1.32,25.26)**	**0.02**
Not had sex	−0.33	0.72	(0.07,7.59)	0.78
**Did you use condoms (recipent)**				0.20
Not 100% condoms		1.00		
100% condoms	0.09	1.10	(0.25,4.75)	0.90
Not had sex	1.42	4.12	(0.75,22.47)	0.10
**Use antibiotics for other purposes**	2.02	7.53	**(2.39,23.64)**	**0.01**
**Have you used mouthwash**	−0.36	0.70	(0.23,2.15)	0.53
**Constant**	−2.86	0.06		0.00

*In past 7 days. The significant of bold values *p* < 0.05.

## 4 Discussion

In this prospective cohort study, we assess chlamydia and gonorrhea infection and spontaneous clearance among MSM in Zhuhai, China. 151 MSM were enrolled, among whom 33.77% tested positive for chlamydia and/or gonorrhoea. Our findings suggest that the rectum is the most susceptible site for infection among MSM. Notably, we observed that 63.5% of all infections cleared spontaneously. Our results indicated that sexual acts, such as kissing male partners, engaging in receptive oral sex, insertive anal sex, and receptive rimming, were associated with increased risk for both chlamydia and gonorrhoea infections, as was being HIV positive. Conversely, using condoms 100% of the time during sex as an insertive partner and having used antibiotics for other purposes in the past 7 days were linked with higher likelihoods of spontaneous clearance. We also found that ethnicity (other than Han), kissing male partners, and not using condoms 100% of the time during sex were associated with increased risk for chlamydia infection, while age was found to be a protective factor. When it comes to gonorrhoea infection, kissing male partners was identified as a risk factor, while younger age was protective. Overall, our study sheds light on the factors that may contribute to chlamydia and gonorrhoea infections and clearance in the MSM population.

Our study identified a higher prevalence of chlamydia and gonorrhoea infections (33.7%) compared to other studies. For instance, a recent study conducted by Chang et al. in Shenzhen reported the prevalence of chlamydia and gonorrhoea among clinic attendees as 9.2 and 2.6%, respectively, in 2020 (15). The higher prevalence in our study may be due to our focus on the MSM population, which is a high-risk group for STIs. Previous studies in Europe, North America, and Australia have reported rectal chlamydia and gonorrhoea infections prevalence of 6–9% and 2–7%, respectively, among MSM (16). Our cohort study design enabled the accurate calculation of incidence rates compared to cross-sectional studies. Additionally, the Pearl River Delta region has seen experienced a significant population influx, which may have contributed to the high incidence of STIs. Moreover, our study reveals a higher prevalence of rectal infection with CT and NG, which is consistent with previous studies conducted in different regions of the world, and provides evidence supporting the idea that rectal infection is more persistent than oral and urethral infections (17).

Kissing has been identified as a significant factor in chlamydia and/or gonorrhoea infection in our study. Previous research has indicated that kissing is the most frequently engaged in sexual activity among MSM (18, 19). Many studies have explored whether kissing with male partners is associated with chlamydia and gonorrhoea infection in MSM. For example, Templeton et al. found that wet kissing (with tongue insertion) among MSM was associated with gonorrhoea in univariate analysis, but this relationship was not significant in multivariate analysis (20). However, Cornelisse et al. noted that due to the high correlations between kissing, fellatio, and rimming, it was difficult to determine the contribution of kissing to gonorrhoea infection in their adjusted analysis (21). The persuasiveness of these two cross-sectional studies is limited. A 12-week cohort study by Chow et al. discovered that the incidence rate ratio of gonorrhea infection increased as the number of kissing partners increased (22).

Our study demonstrates a similar spontaneous clearance rate of CT/NG infections compared with previous studies (12). Additionally, our findings show that consistent condom usage during sexual encounters can prevent chlamydia infection and promote natural clearance. A prospective cohort study conducted in Abuja and Lagos, Nigeria (5) also confirmed that correct condom use during every sexual encounter is crucial to prevent the spread of various STIs, including HIV, among MSM. Therefore, multiple strategies should be explored to promote condom use in MSM populations in the future. Our study identified receptive oral sex, insertive anal sex, and receptive rimming were identified as risk factors for chlamydia and gonorrhea infection. Chow et al. found that an increased number of penile-oral sex partners was associated with gonorrhea infection, but not with an increased number of insertive rimming partners (22). However, a previous cross-sectional study suggested that rimming played a significant role in gonorrhea transmission among MSM, without distinguishing between insertive and receptive rimming (23). Our study, on the other hand, categorized sexual behaviors into receptive or insertive, as well as oral sex, anal sex, and rimming, allowing us to identify which specific sexual behaviors were risk factors for chlamydia and/or gonorrhea infection.

Our study found that the risk of developing gonorrhea infection in MSM was inversely related to age, and this is consistent with previous studies (24–26). This suggests that younger MSM may engage in more unsafe sexual practices due to a lack of knowledge and awareness of STI prevention, which increases their susceptibility to NG. Therefore, it is crucial to concentrate on enhancing STI-related health education for young MSM and improving NG screening among this group. Additionally, age was found to be a protective factor for spontaneous clearance of NG, which may be due to younger MSM generally having better overall health and more robust immune systems that can effectively clear the pathogen.

This study has several limitations. Firstly, self-administered questionnaires were used to assess participants’ sexual behaviors, which may have protected their privacy but could have led to an underestimation of the actual number of risky behaviors, resulting in biased reporting of sexual behaviors. Secondly, although we adjusted for confounding variables, there may still be residual confounders that were not measured, such as bacterial load or genotype, which could affect the spontaneous clearance of NG and CT, or help to determine whether multiple co-infections originated from the same bacteria. Thirdly, MSM have strong sexual demands, but we only collected information on their recent sexual behavior steps during the follow-up and baseline questionnaires, which may not be representative of their overall sexual behavior. Additionally, MSM may engage in different types of sexual behaviors with different types of partners, which could lead to varying infection risks. However, we did not differentiate between permanent and temporary partners. Furthermore, the sexual behaviors we included in this study were limited, and other types of sexual behaviors, such as the use of massage sticks or multiple sexual intercourse, could have influenced the results.

## 5 Conclusion

Our study reveals a high prevalence of CT and NG infections among MSM, particularly in the rectal area. The findings suggest that engaging in specific sexual behaviors increases the risk of CT or NG infection, with having an HIV infection, kissing a partner, and performing oral sex on a male partner in the past week being risk factors. Age and consistent condom use were protective factors against CT and NG infection. More than half of patients experienced spontaneous clearance. Our analysis indicates that younger age and certain sexual behaviors were influence factors of infection and spontaneous clearance. It is advisable to enhance STI screening and the importance of promoting safe sexual practices, particularly among young MSM.

## Data availability statement

The original contributions presented in the study are included in the article/supplementary material, further inquiries can be directed to the corresponding authors.

## Ethics statement

The studies involving humans were approved by the study protocol was approved by the Institutional Review Board (No. 2020–1289) of Xi’an Jiaotong University. The studies were conducted in accordance with the local legislation and institutional requirements. The participants provided their written informed consent to participate in this study.

## Author contributions

HL: Writing – original draft, Project administration, Writing – review & editing. HT: Data curation, Writing – review & editing. YF: Data curation, Methodology, Validation, Writing – review & editing. ShuH: Methodology, Writing – review & editing. YW: Writing – review & editing. LaZ: Supervision, Writing – review & editing. ShaH: Supervision, Writing – review & editing. JL: Validation, Writing – review & editing. HZ: Software, Writing – review & editing. XH: Project administration, Supervision, Validation, Writing – review & editing. WT: Writing – review & editing, Supervision, Validation. YZ: Resources, Writing – review & editing. LeZ: Writing – review & editing.
